# Free-breathing high-resolution respiratory-gated radial stack-of-stars magnetic resonance imaging of the upper abdomen at 7 T

**DOI:** 10.1002/nbm.5180

**Published:** 2024-05-22

**Authors:** Ivo T. Maatman, Jenni Schulz, Sjoerd Ypma, Kai Tobias Block, Sebastian Schmitter, John J. Hermans, Ewoud J. Smit, Marnix C. Maas, Tom W. J. Scheenen

**Affiliations:** 1 Department of Medical Imaging, Radboud University Medical Center, Nijmegen, the Netherlands; 2 Erwin L Hahn Institute for MR Imaging, Essen, Germany; 3 Department of Radiology, NYU Langone Health, New York, New York, USA; 4 Physikalisch-Technische Bundesanstalt (PTB), Braunschweig, Berlin, Germany

**Keywords:** radial sampling, respiratory gating, TIAMO, ultrahigh field MRI

## Abstract

Ultrahigh field magnetic resonance imaging (MRI) (≥ 7 T) has the potential to provide superior spatial resolution and unique image contrast. Apart from radiofrequency transmit inhomogeneities in the body at this field strength, imaging of the upper abdomen faces additional challenges associated with motion-induced ghosting artifacts. To address these challenges, the goal of this work was to develop a technique for high-resolution free-breathing upper abdominal MRI at 7 T with a large field of view. Free-breathing 3D gradient-recalled echo (GRE) water-excited radial stack-of-stars data were acquired in seven healthy volunteers (five males/two females, body mass index: 19.6–24.8 kg/m^2^) at 7 T using an eight-channel transceive array coil. Two volunteers were also examined at 3 T. In each volunteer, the liver and kidney regions were scanned in two separate acquisitions. To homogenize signal excitation, the time-interleaved acquisition of modes (TIAMO) method was used with personalized pairs of B_1_ shims, based on a 23-s Cartesian fast low angle shot (FLASH) acquisition. Utilizing free-induction decay navigator signals, respiratory-gated images were reconstructed at a spatial resolution of 0.8 × 0.8 × 1.0 mm^3^. Two experienced radiologists rated the image quality and the impact of B_1_ inhomogeneity and motion-related artifacts on multipoint scales. The images of all volunteers showcased effective water excitation and were accurately corrected for respiratory motion. The impact of B_1_ inhomogeneity on image quality was minimal, underscoring the efficacy of the multitransmit TIAMO shim. The high spatial resolution allowed excellent depiction of small structures such as the adrenal glands, the proximal ureter, the diaphragm, and small blood vessels, although some streaking artifacts persisted in liver image data. In direct comparisons with 3 T performed for two volunteers, 7-T acquisitions demonstrated increases in signal-to-noise ratio of 77% and 58%. Overall, this work demonstrates the feasibility of free-breathing MRI in the upper abdomen at submillimeter spatial resolution at a magnetic field strength of 7 T.

## 1 │ INTRODUCTION

Magnetic resonance imaging (MRI) at a magnetic field strength of 7 T has the potential to enable novel applications for noninvasive imaging of the human body. Because of the inherent increase in sensitivity,^1^ imaging can be conducted at high spatial resolutions. In addition, shorter T_2_^*^ and longer T_1_ relaxation times at ultrahigh field (UHF) enable capturing different or stronger image contrasts than available at clinically used field strengths.^2^

Prior research has demonstrated multiple uses of 7-T MRI across diverse body regions. Compared with 1.5 and 3 T, the increased magnetic field strength allows a superior contrast-to-noise ratio (CNR) in brain functional MRI (fMRI), enabling the detection of weaker effects, improved functional mapping accuracy, and enhanced spatial resolution.^3,4^ fMRI at submillimeter resolution has been feasible exclusively with UHF strengths.^4^ In many neurological and brain disorders a clinical role of MRI at 7 T has been described, for example, in multiple sclerosis,^5^ Parkinson’s disease,^6^ epilepsy,^7^ intracranial aneurysms,^8^ and myelopathies.^9^ As well as brain applications, 7 T also shows potential for imaging the abdomen.^3,10^ High-resolution 7-T renal MRI demonstrated increased corticomedullary distinction,^11^ which may allow superior differentiation between renal tumor types^12^ and facilitate evaluation of allograft function in kidney transplant patients.^13^ Additionally, hyperintense renal and liver vasculature in 7-T images has shown promise for MR angiography without contrast agents.^14^ This could be advantageous for patients with impaired renal function, for whom gadolinium-based contrast agents are contraindicated because of the increased risk of nephrogenic systemic fibrosis.^15,16^ For prostate cancer in combination with iron oxide nanoparticles, 7-T MRI allowed the detection of smaller lymph nodes, suspected of harboring metastases, than at 3 T using similar acquisition times, providing better informed treatment and surgical planning.^17,18^ Similarly, improved characterization of lymph nodes in the upper abdomen with nanoparticle-enhanced MRI at 7 T would increase the accuracy of disease staging for patients with cancers metastasizing to this region, such as esophageal, pancreatic, gastric, renal, or cervical cancers.^19^

In nearly all UHF studies in the body to date, a multitransmit coil array was employed to homogenize the signal distribution across the imaged volume.^15,17,20–23^ Because the wavelength of radiofrequency (RF) pulses decreases with increasing magnetic field strength, the transmit field $B_{1}^{+}$ exhibits a strong spatial dependence.^24,25^ To prevent $B_{1}^{+}$ dropouts or harmful specific absorption rate (SAR) levels due to the RF interference of the different transmit channels, RF shimming or parallel transmit (pTx) techniques with multiple transmit elements are frequently applied.^26^ In the case of pTx, each coil element transmits its own channel-specific waveform that includes the carrier phase, amplitude, and pulse envelope per transmit channel.^27–29^ Utilizing pTx offers numerous degrees of freedom, granting flexibility in distributing flip angles across the imaged volume. However, this increased flexibility comes with trade-offs, such as a time-consuming calibration process, relatively long pulse durations, and susceptibility to off-resonance effects, particularly in the presence of motion.^30,31^ RF shimming methods, on the other hand, are typically easier to implement because they only adjust the phases and amplitudes across channels while the underlying pulse envelopes remain identical.^32,33^ However, the performance of RF shimming is generally limited when utilized for applications with a large field of view (FOV).

A technique known as time-interleaved acquisition of modes (TIAMO) offers an intermediary approach between RF shimming and pTx. This technique is particularly well suited for large FOV imaging without requiring an overly intricate RF pulse design.^34^ Using TIAMO, each k-space line is acquired multiple times with two different $B_{1}^{+}$ phase shim settings with complementary spatial $B_{1}^{+}$ distributions. The 2-fold increase in acquisition time can be compensated by considering each second acquisition of the same k-space line as an additional set of (virtual) receivers, enabling the use of increased parallel imaging factors.

Although the effectiveness of TIAMO for large FOV imaging has been shown in multiple previous studies of 7-T body MRI,^17,18,34^ imaging results in the upper abdomen tend to be compromised by respiratory motion.^35,36^ At lower field strength, artifacts resulting from respiratory motion have been successfully mitigated using free-breathing non-Cartesian k-space acquisitions. One widely adopted non-Cartesian acquisition to reduce motion artifacts is the radial stack-of-stars 3D gradient-recalled echo (GRE) sequence.^37^ The radial stack-of-stars sequence acquires k-space as stacks of intersecting radial projections, leading to cylindrical k-space coverage. When combined with a golden-angle readout scheme, radial stack-of-stars data can be retrospectively sorted into different respiratory phases based on surrogate motion signals to minimize motion blur.^38–41^ The non-Cartesian k-space trajectory ensures robustness against ghosting artifacts, while its single Cartesian phase-encoding axis maintains signal-to-noise ratio (SNR) efficiency. Because the sequence traces out straight lines in k-space, it demonstrates reduced sensitivity to gradient errors compared with other SNR-efficient non-Cartesian sequences employing dynamic waveforms.

Despite regular use of radial stack-of-stars sequences at 3 T, the feasibility for upper abdominal MRI at 7 T has not yet been investigated. Non-Cartesian sequences have generally received limited attention at UHF strengths because of their sensitivity to susceptibility, field inhomogeneity, chemical shift, and gradient-delay artifacts.^14,42^ Gradient-delay errors are generally modeled as being independent of the main field strength.^43^ However, the magnitudes of susceptibility and off-resonance errors are proportional to the strength of the main field,^44^ making non-Cartesian MRI more challenging than its Cartesian counterpart.

In this study, we demonstrate the feasibility of free-breathing MRI of the abdomen at 7 T using a golden-angle radial stack-of-stars sequence in healthy volunteers at a high spatial resolution. To prevent $B_{1}^{+}$ artifacts, dual-mode phase-only RF-shimming was performed robustly using the TIAMO technique. To ensure accurate motion correction, even when acquiring many partitions and multiple TIAMO modes, we employed the “single-spoke binning” method, which utilizes free induction decay (FID) navigators and a randomized partition sampling order.^41^ To prevent the off-resonance blurring caused by lipid signal, RF excitation was carried out using a water-excitation pulse. Data analysis included quantitative measures of image homogeneity, a qualitative radiological evaluation of small anatomical structures, and a paired comparison with 3-T acquisitions in a subset of volunteers.

## 2 │ METHODS

### 2.1 │ $B_{1}^{+}$ calibration

A custom body-array coil, consisting of eight fractionated dipole antennas,^45^ was employed for transmission and reception of the RF signal. The pTx coil was fully integrated into the 7-T MR system, including a vendor-supplied virtual observation points file for local SAR supervision. Relative $B_{1}^{+}$ mapping was performed to optimize two complementary TIAMO shim modes in a subject-specific manner using a single-slice 2D Cartesian GRE sequence in a free-breathing acquisition over 23 s. The sequence consecutively transmitted on each of the eight channels and received signals on all channels. Provided that the dipole antennas are oriented parallel to the head–feet direction and given a maximum slab thickness of 16 cm (Table 1), the $B_{1}^{+}$ fields could be approximated to be constant within the FOV along the slab direction.^45^ The calculations for obtaining relative $B_{1}^{+}$ maps from the resulting data followed the descriptions in Van der Moortele et al. and Dietrich et al.^46,47^

To homogenize the signal distribution using TIAMO, optimal sets of RF phases were determined using a genetic algorithm.^48^ The fitness function *F* used was the sum-of-squares difference in image magnitude, evaluated within a manually selected region of interest (ROI), which covered the body contour:

(1) $$F=\sum_{r\in \text{ROI}}{\left(\frac{g\left(\text{r}\right)}{\mu }-1\right)}^{2},$$

with scalar μ corresponding to the mean of $g\left(\text{r}\right)$ within the ROI and $g\left(\text{r}\right)$ defined as

(2) $$g\left(\text{r}\right)=\sqrt{\sum_{m}{\left|\sum_{k}B_{1k}^{+}\left(\text{r}\right)e^{i\phi_{mk}}\right|}^{2}}.$$

Here, $B_{1k}^{+}\left(\text{r}\right)$ denotes the vectorized transmit map for channel k obtained using the calibration scan. Each $\varphi_{mk}$ represents a variable transmit RF phase of TIAMO mode *m* and transmit channel k. The optimal sets of RF phases were selected as those that minimize Equation (1) within 150 iterations using two TIAMO modes. The minimization was performed offline in MATLAB (MathWorks, Natick, MA, USA).

### 2.2 │ Radial stack-of-stars sequence

**FIGURE 1:**
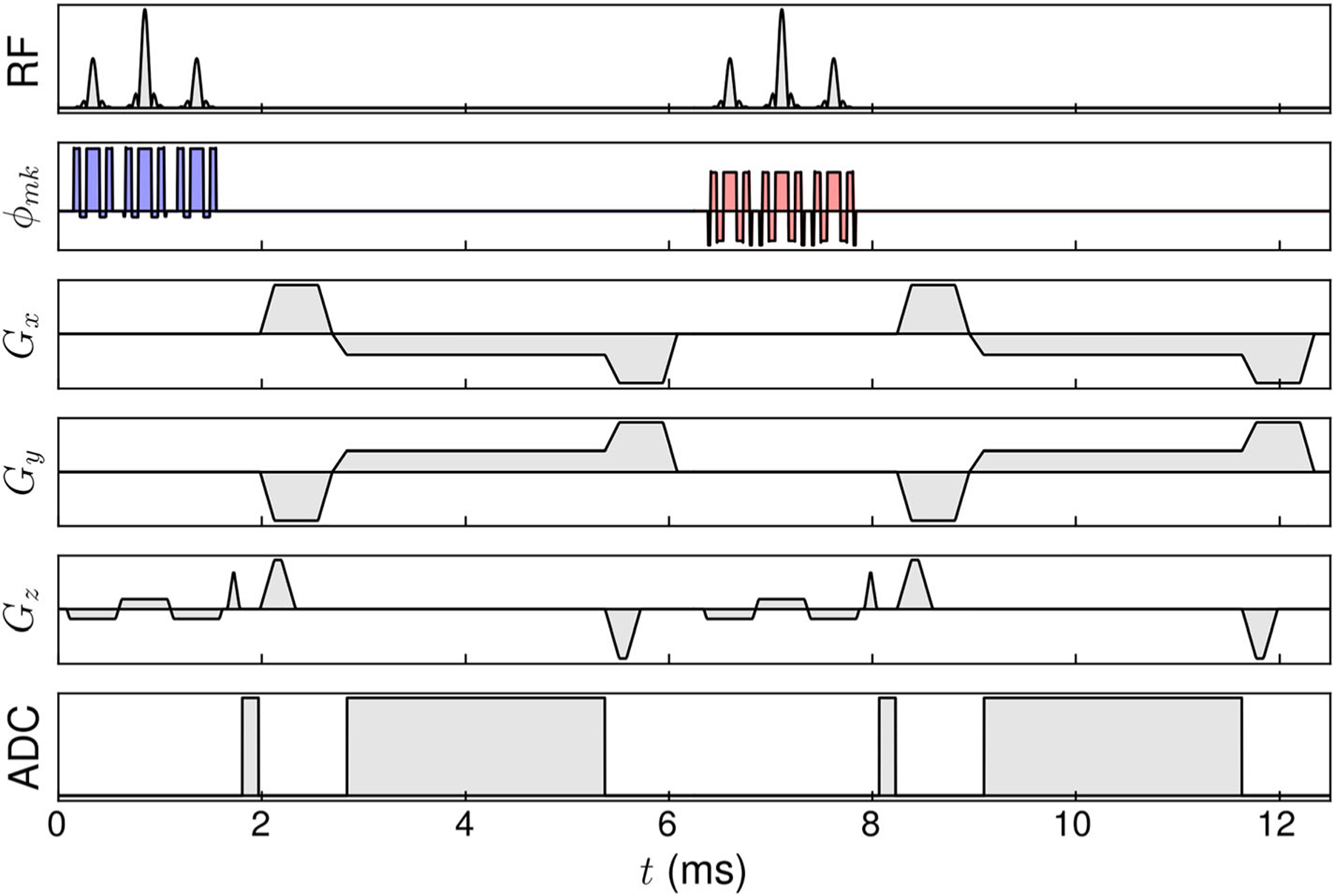
Diagram of the golden-angle radial stack-of-stars spoiled gradient-recalled echo pulse sequence with TIAMO $B_{1}^{+}$ shim. The phases of the two TIAMO modes for a single transmit channel are indicated in blue and red. The complementary excitation modes were identical in spatial encoding, differing only in the phases used for the RF pulses. Slab-selective water excitation was performed using a (121) binomial pulse with bipolar gradients. A nonlocalized FID acquisition between slab-selection rephasing and partition encoding acted as a respiratory navigator. ADC, analog-to-digital converter; FID, free induction decay; RF, radiofrequency; TIAMO, time-interleaved acquisition of modes.

**FIGURE 2:**
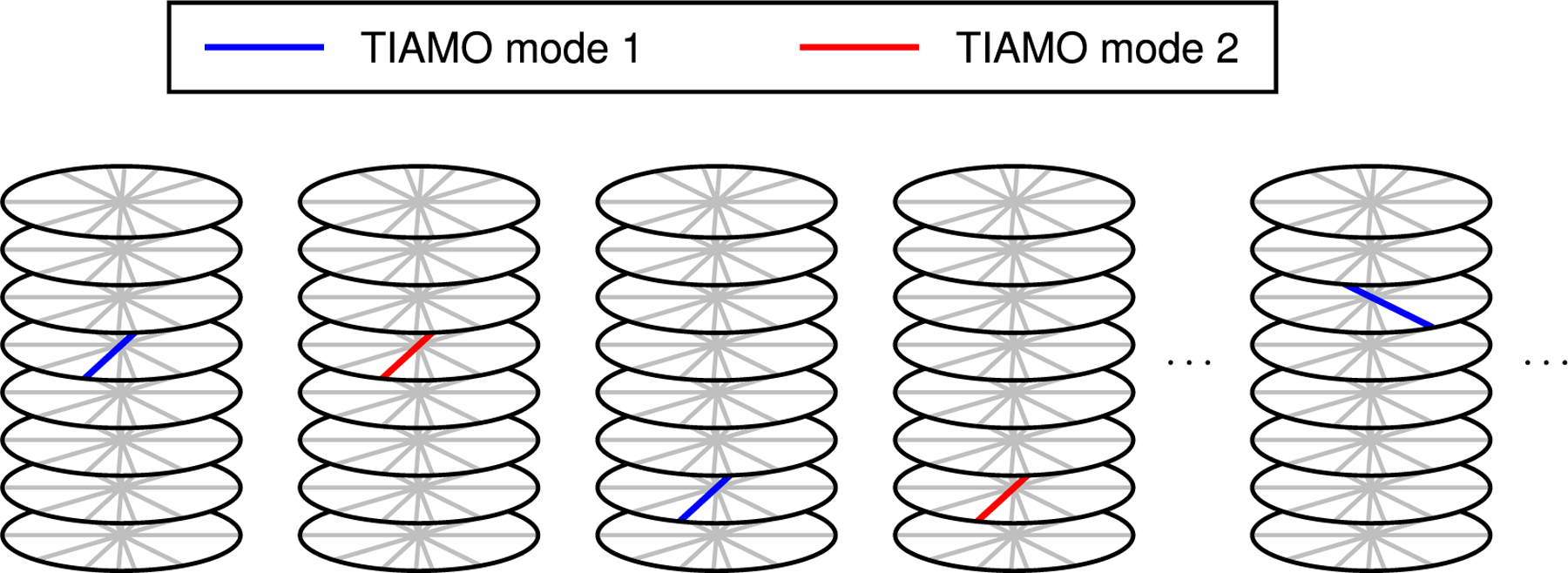
Acquisition scheme of the radial stack-of-stars sequence (image inspired by Wang et al.^50^). Using TIAMO, each readout was acquired with two complementary RF shim modes (blue and red lines). All phase-encode steps were scanned twice, once per spoke direction and TIAMO mode, while using a randomized partition acquisition order according to the single-spoke binning method. After scanning an entire spoke stack, the readout angle was incremented by the golden angle and the process was repeated for the updated projection angle. RF, radiofrequency; TIAMO, time-interleaved acquisition of modes.

The radial sequence used in this work was a modified version of the 3D golden-angle radial stack-of-stars GRE sequence described in^37,41^ (Figure 1). A slab-selective water-excitation pulse was incorporated into the sequence to avoid off-resonance blurring of lipid signal. This (121) binomial pulse had a total duration of 1.53 ms with a 75% duty cycle and used sinc-shaped subpulses with a time-bandwidth product of 7. It was executed with bipolar instead of monopolar gradients to maximize the duty cycle of RF transmission (see on 4).^49^ The two TIAMO modes were acquired consecutively with identical spatial encoding before advancing to the next phase-encode step or radial view angle, differing only in the RF phases used for the transmit channels (Figure 2). The sequence was gradient-spoiled and RF-spoiled with a *ψ* = 50^°^ quadratic phase increment applied according to the recursive formula^51^:

(3) $$\varphi_{j+1}=\varphi_{j}+j\psi$$

Here, index j counts the RF excitations and is also incremented between the complementary TIAMO modes.

An FID navigator acquisition consisting of 32 datapoints sampled within 200 *μ*s was inserted between the excitation and phase-encoding gradients of each TR to extract respiratory signals for retrospective motion correction. In contrast to common self-gating techniques,^38,39,52^ the accuracy of respiratory signal obtained through the FID readouts is independent of the numbers of phase-encode steps and TIAMO modes. Therefore, even with a sampling duration of up to 825 ms for each spoke angle, respiratory signals could still be accurately detected with a temporal resolution of 12.5 ms, significantly below the recommended threshold of 400 ms for accurate self-gating.^53^ All partition-encoding steps were acquired for every spoke angle before switching to the next angle, where the acquisition order of partitions was randomized without replacement to ensure an even distribution of readouts across partitions after respiratory sorting.^41^ A golden-angle rotation of 111.25^°^ was applied between subsequent spoke stacks.

### 2.3 │ Measurements

MR data were acquired in seven volunteers (five males/two females, age: 26–49 years, body mass index [BMI]: 19.6–24.1 kg/m^2^) using the golden-angle radial stack-of-stars sequence (Table 1) on a 7-T MRI system (MAGNETOM Terra; Siemens Healthineers, Erlangen, Germany) with an eight-channel body array coil. This volunteer study was conducted with approval of the local Institutional Review Board and written informed consent was obtained from all volunteers. Data were acquired of the liver and kidney region of each volunteer in two separate radial scans with an acquired spatial resolution of 0.8 × 0.8 × 2.0 mm^3^ (Table 1). To minimize artifacts due to gradient nonlinearities, all acquisitions were carried out in axial orientation.^54,55^ A readout bandwidth of 390 Hz/px allowed for a relatively long acquisition window within one TR, increasing the SNR efficiency of the acquisition. Gradient calibration was utilized to correct for gradient delays.^56^

To remain within the SAR limits, with a TR of 6.25 ms, the maximum permitted voltage of the binomial pulse in the radial acquisition was 65 V. We used this fixed maximum voltage for the excitation pulse for all volunteers. In the absence of absolute $B_{1}^{+}$ mapping in the individual volunteer examinations, this resulted in some variability in the actual flip angle between volunteers. To provide an approximate flip angle, two exhale breath-hold 3D dual refocusing echo acquisition mode (3DREAM)^57^ acquisitions were performed in a single volunteer, with two complementary TIAMO shim modes optimized according to Equation (1). The parameters of the 3DREAM acquisitions included TE_STE_ = 1.07 ms, TE_FID_ = 2.14 ms, TR = 7.9 s, FOV = 350 × 350 mm^2^, slice thickness = 6 mm, matrix size = 60 × 60 × 40, and bandwidth = 1005 Hz/px.

To compare results obtained at 7 T with a clinical standard, liver data were obtained at 3 T (MAGNETOM Prisma Fit; Siemens Healthineers) for two of the seven volunteers using an optimized protocol. Both 3-T measurements were performed using standard 32-channel spine and 18-channel body array coils. The scan parameters closely matched those of the 7-T measurements (Table 1). However, because of the lower resonance frequency at 3 T, TE and TR were slightly longer because of a prolonged binomial pulse duration of 3.43 ms. Nevertheless, because no TIAMO shim was required, the 3-T measurement allowed for a larger number of radial views in the same measurement time.

### 2.4 │ Reconstruction

The k-space data from the two complementary TIAMO modes were processed as if they had been collected as a single signal average using twice the number of receiver elements: we concatenated the data array of the second TIAMO mode as eight additional coil elements to the first array of data of eight coil elements. The resulting 16 coil images were reconstructed offline with 3D motion-averaged or 4D respiratory-gated inverse nonuniform fast Fourier transforms (iNUFFTs). To this end, k-space data were weighted according to the sampling density and resampled onto a rectilinear grid, followed by a 3D Cartesian inverse Fast Fourier Transformation.

To enable accurate respiratory gating even when acquiring a large number of partitions and two TIAMO modes, readouts were sorted into motion phases according to the single-spoke binning technique.^41^ Therefore, respiratory signals with temporal resolution of 2 TR were estimated from the FID navigator data. Assuming that no appreciable respiratory motion occurs within two TRs (≈12 ms), it was ensured that complementary k-space lines from the two subsequent TIAMO readouts were assigned to the same motion phase. Consequently, navigator samples from the two complementary TIAMO modes were processed in the same way as the k-space data, that is, by combining the data into 16 (virtual) channels. Furthermore, the first and last five sample points per FID readout were discarded because of systematic errors, and the magnitudes of the remaining samples were averaged. Time-curve artifacts were resolved through the subtraction of a baseline eddy current signal,^58^ while a low-pass filter eliminated frequency components beyond the range associated with respiratory motion (> 2 Hz). Different respiratory signals from each (virtual) channel were combined using principal component analysis. To address baseline drifts in the self-gating signal, a cubic spline interpolation was performed between the maximal exhalation points to estimate a correction signal.^59^

To prevent motion artifacts in the final reconstructions, 40% of the data nearest to the end-expiratory phase were selected for reconstruction with iNUFFTs according to the extracted respiratory signal.^53,60^ To investigate the effect of using different ratios of data for the respiratory-gated reconstruction, a radiologist (JJH) evaluated four separate reconstructions of a single volunteer using 25%, 40%, 50%, or 66% of the total data (Figure S1). The acceptance ratio of 40% was selected for all reconstructions, being considered the most suitable trade-off between motion blur and residual undersampling.

Complex-valued coil sensitivity profiles were estimated for each of the 16 (virtual) receivers from low-resolution iNUFFT reconstructions of the 3D motion-averaged data to perform $B_{1}^{-}$ -weighted coil combinations. Recombined images were weighted with a filter that reduced inhomogeneity caused by the receiver sensitivity profiles.^61^

Reconstructions of 3-T data, intended for paired comparison with images resulting from the 7-T scans, were performed in a similar fashion to those at UHF: respiratory signals were generated from FID navigators and motion-gated reconstructions were performed utilizing a 40% acceptance ratio.

### 2.5 │ Data analysis

Image homogeneity of the default, circularly polarized (CP) RF shim and the optimized TIAMO shim were evaluated using coefficients of variation, similar to the descriptions in^47,62^:

(4) $$\text{CV=}\frac{\text{std}\left(\sum_{m}\left|\sum_{k}B_{1k}^{+}e^{i\phi mk}\right|\right)}{\text{mean}\left(\sum_{m}\left|\sum_{k}B_{1k}^{+}e^{i\phi mk}\right|\right)},$$

with std denoting the standard deviation.^47,62^ Furthermore, the transmit efficiencies were calculated as^47,63^:

(5) $$\varepsilon =\text{mean}\left(\frac{\sum_{m}\left|\sum_{k}B_{1k}^{+}e^{i\phi mk}\right|}{m\sum_{k}\left|B_{1k}^{+}\right|}\right),$$

with $m\in \left\{1\right\}$ and $m\in \left\{1,2\right\}$ for the CP and TIAMO shims, respectively. Equation (5) describes the ratio between the signal intensity provided by the shim and the maximum signal intensity achievable under complete phase coherence across all channels. The above calculations were performed using 50 × 90 pixels ROIs in the reconstructions of the Cartesian GRE $B_{1}^{+}$ calibration data. Levels of statistical significance were determined via Wilcoxon signed-rank tests with p values of less than 0.05 deemed significant.

[Correction added on 27 May 2024, after first online publication: the subscript ‘m’ has been added in both Greek phi of Equation (4).]

All images were qualitatively analyzed independently by two radiologists (JJH and EJS, with 25 and nine years of experience in reading abdominal MRI examinations, respectively). Image quality regarding clinically relevant details of the liver and kidney datasets was rated on a five-point scale (i.e., 1: not visible or nondiagnostic; 2: poor; 3: moderate; 4: good; 5: excellent). Ratings were performed separately for axial, coronal, and sagittal image orientations. The median and interquartile ranges (IQRs) of the Likert-scale scores were calculated for each of the following factors. Evaluation of the kidney datasets was focused on (i) depiction of the adrenal glands; (ii) the proximal ureter; and (iii) the renal veins. All liver datasets were evaluated on (i) depiction of the liver vessels; and (ii) delineation of the diaphragm.

Furthermore, the presence of artifacts within the body was assessed using three categories (1: nondiagnostic due to severe impairment; 2: moderate impairment; 3: no or insignificant impairment). The following artifacts were evaluated: (i) ${\text{B}}_{1}$ inhomogeneity; and (ii) motion artifacts. Impairments resulting from ${\text{B}}_{1}$ inhomogeneity were analyzed considering both the extent of signal dropouts and their respective locations. The evaluation of ${\text{B}}_{1}$ inhomogeneity scores also encompassed limitations in RF penetration depth and some residual weighting due to coil receiver sensitivity, evident through signal loss at the center of the FOV. Similarly, image impairments due to motion included streaking and blurring artifacts. Although other factors besides motion contributed, including a slight undersampling factor of 1.50, it proved to be too challenging to reliably discern the different causes of the streaking artifacts in the qualitative evaluation.

To compare results obtained at UHF with a clinical standard, voxel-wise SNR maps were derived from the paired 3- and 7-T measurements by leveraging an established Monte Carlo method.^64^ This “pseudo multiple replica” method allowed consistent calculations of SNRs while being unaffected by differences in the underlying k-space trajectories. Noise calibration data were obtained before the imaging sequence and consisted of 30 readouts of 768 samples per receive channel without RF transmission or gradient encoding. For the purpose of a single simulation, 50 image replicas were reconstructed. Because of significant phase gradients across the resulting images, SNRs were calculated from the signal and noise magnitudes. To mitigate bias from inconsistent lipid suppression, overall mean SNR differences were calculated across water-only 100 × 70 × 40 voxel ROIs positioned within the liver.

## 3 │ RESULTS

### 3.1 │ Acquisition and reconstruction

**FIGURE 3:**
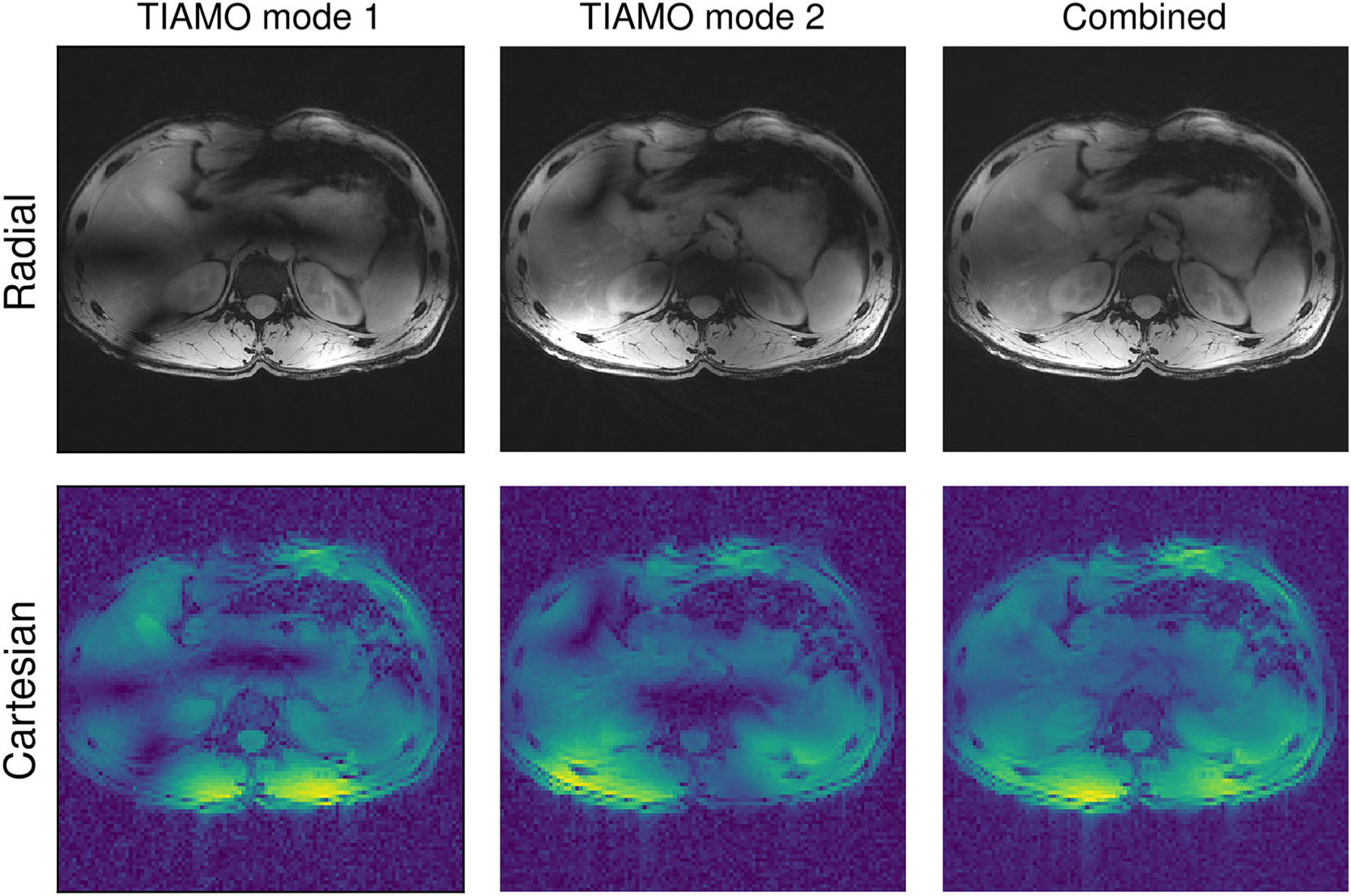
Representative example of a radial stack-of-stars liver acquisition with the TIAMO $B_{1}^{+}$ shim. The top row contains motion-averaged reconstructions of the radial data for both TIAMO modes, the right column shows the sum of both. The bottom row shows the magnitude of the complex sums of the $B_{1}^{+}$ maps, modulated by the phase factors per mode obtained from the calibration (according to Equation 2). TIAMO, time-interleaved acquisition of modes.

**FIGURE 4:**
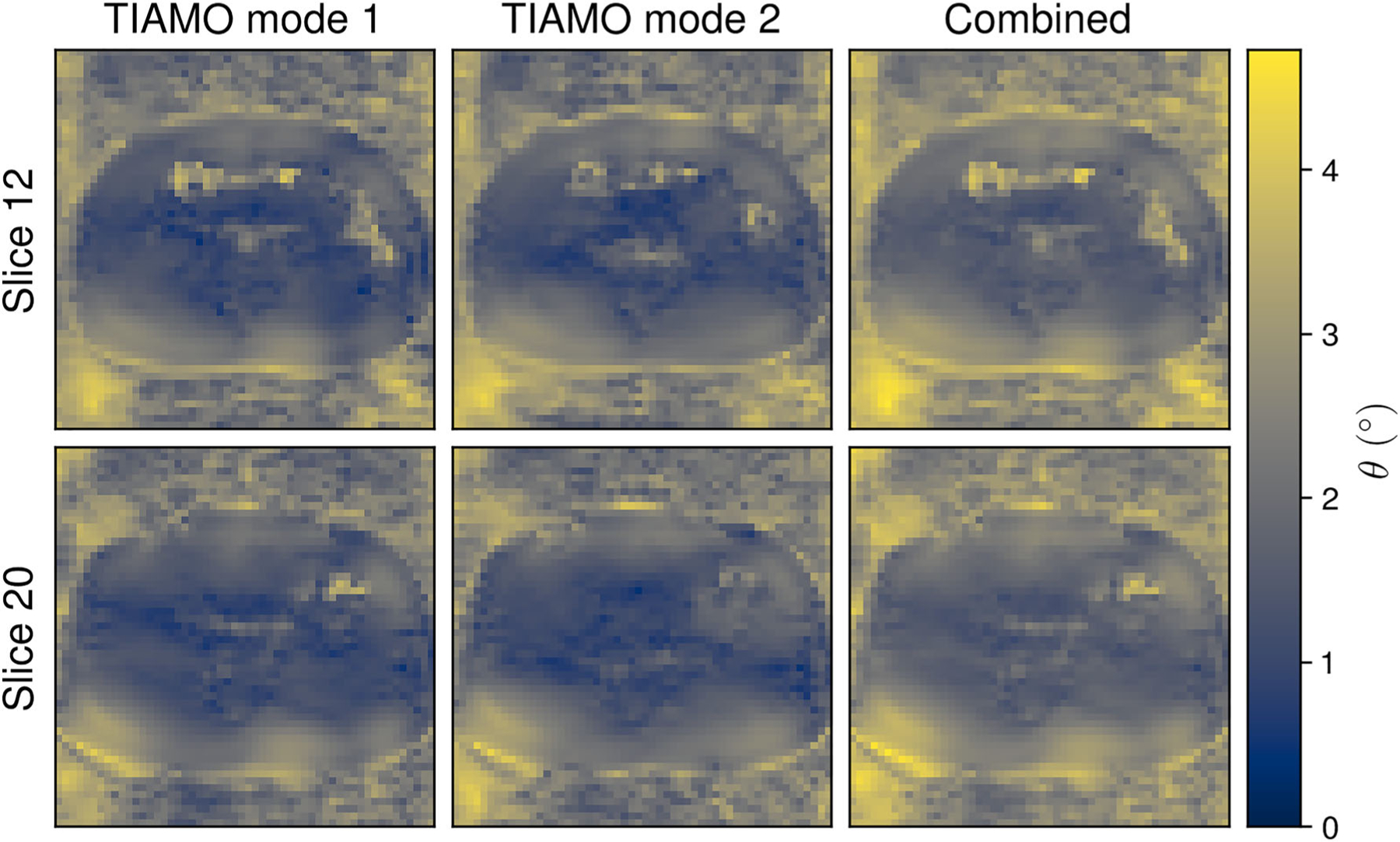
Estimated flip angle distributions of the radial stack-of-stars sequence across two slices in a single volunteer. Flip angle maps were generated by scaling 3DREAM flip angle maps according to the integral of the radial sequence’s binomial pulse RF envelope. 3DREAM, 3D dual refocusing echo acquisition mode; RF, radiofrequency.

The use of TIAMO greatly improved signal homogeneity by effacing the visible signal dropouts in separate reconstructions of the two RF excitation modes (Figure 3). Mean coefficients of variation in all Cartesian calibration datasets significantly decreased ( *p* = 0.0020), with values of 0.62 ± 0.04 (mean ± STD) and 0.42 ± 0.06 for the single CP mode and the combined optimized TIAMO modes, respectively. Some residual signal loss was evident in the combined images acquired through the radial sequence (Figure 3). The residual inhomogeneity was reduced in the final image reconstructions by applying the aforementioned receive sensitivity filter.^61^ Meanwhile, differences in transmit efficiency were insignificant ( *p* = 0.16), with values of 0.46 ± 0.02 and 0.49 ± 0.03 for the CP and TIAMO methods, respectively. This outcome is due to the large FOV, which, for RF-shimming techniques, compromises transmit efficiency.^33,63^ According to the two 3DREAM acquisitions, the mean flip angle allowed in the short-TR radial sequence was estimated at 2.5 ± 0.8^°^ (Figure 4).

**FIGURE 5:**
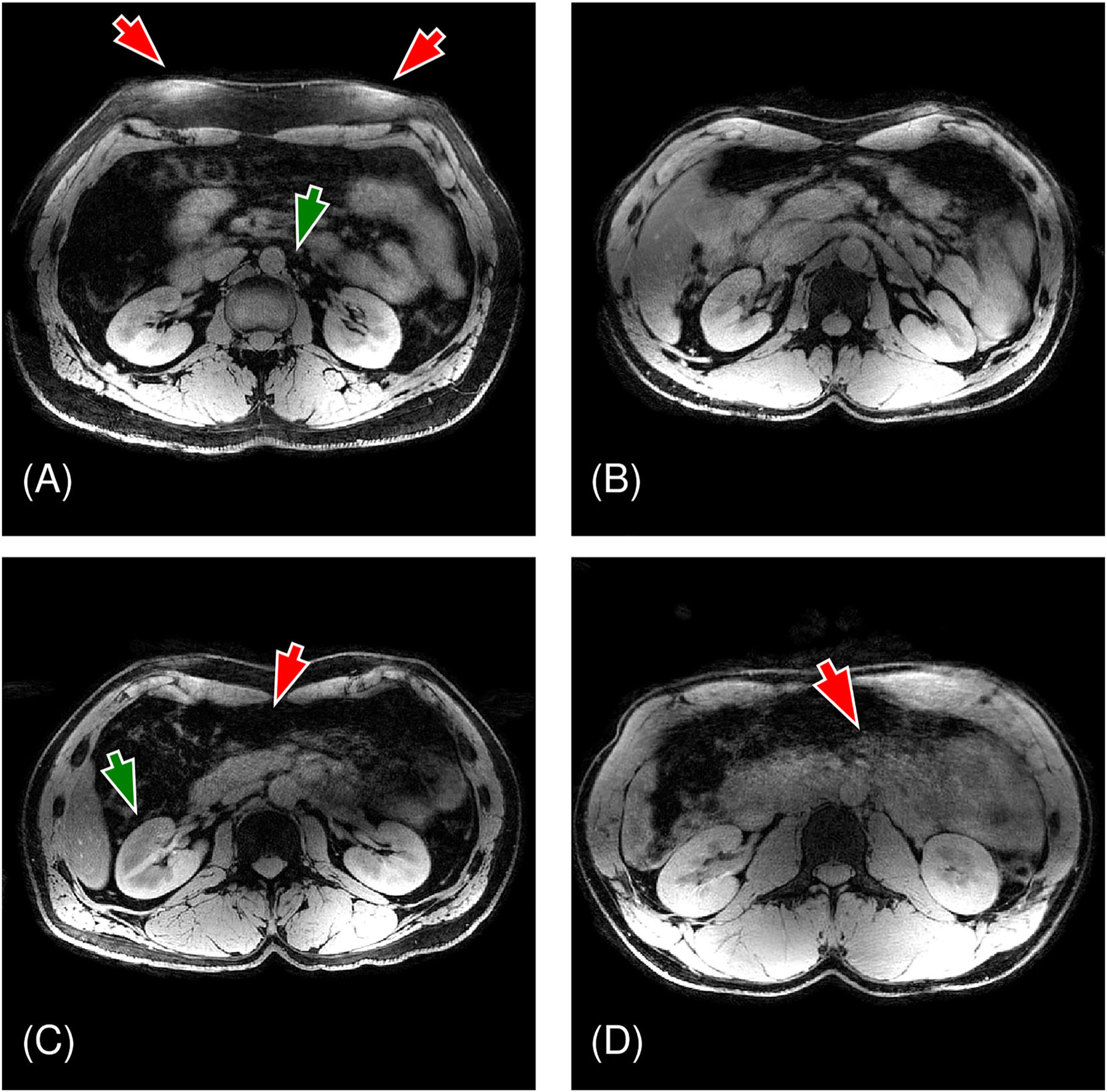
(A–D) Axial views of kidney MRI data of four volunteers. The images illustrate the effectiveness of the TIAMO shim and water excitation. The images show a sharp depiction of the kidneys, vessels, and lymph nodes (green arrow in (A)), as well as clear corticomedullary distinction (green arrow in (C)). Some anterior subcutaneous fat signal remained (red arrows in (A)), likely due to B_o_ inhomogeneities in areas strongly affected by respiratory motion. Magnetic susceptibility differences led to signal loss at the intestines (red arrow in (C)), obscuring the intestinal wall. Residual motion caused streaking artifacts on the anterior side (red arrow in (D)). MRI, magnetic resonance imaging; TIAMO, time-interleaved acquisition of modes.

Fat signals were effectively suppressed using the slab-selective binomial water excitation pulse, with only minor residual subcutaneaous lipid signal detected anteriorly in some subjects (Figure 5A). These remaining lipid signals were likely caused by inhomogeneities in the ${\text{B}}_{0}$ field and were more prominently visible in the subjects with higher BMIs. Changes in magnetic susceptibility at air–tissue interfaces led to significant loss due to intravoxel dephasing, obscuring the intestinal wall (Figure 5C). Additionally, a combination of intestinal motion and off-resonance resulted in noticeable streak artifacts on the anterior side (Figure 5D).

**FIGURE 6:**
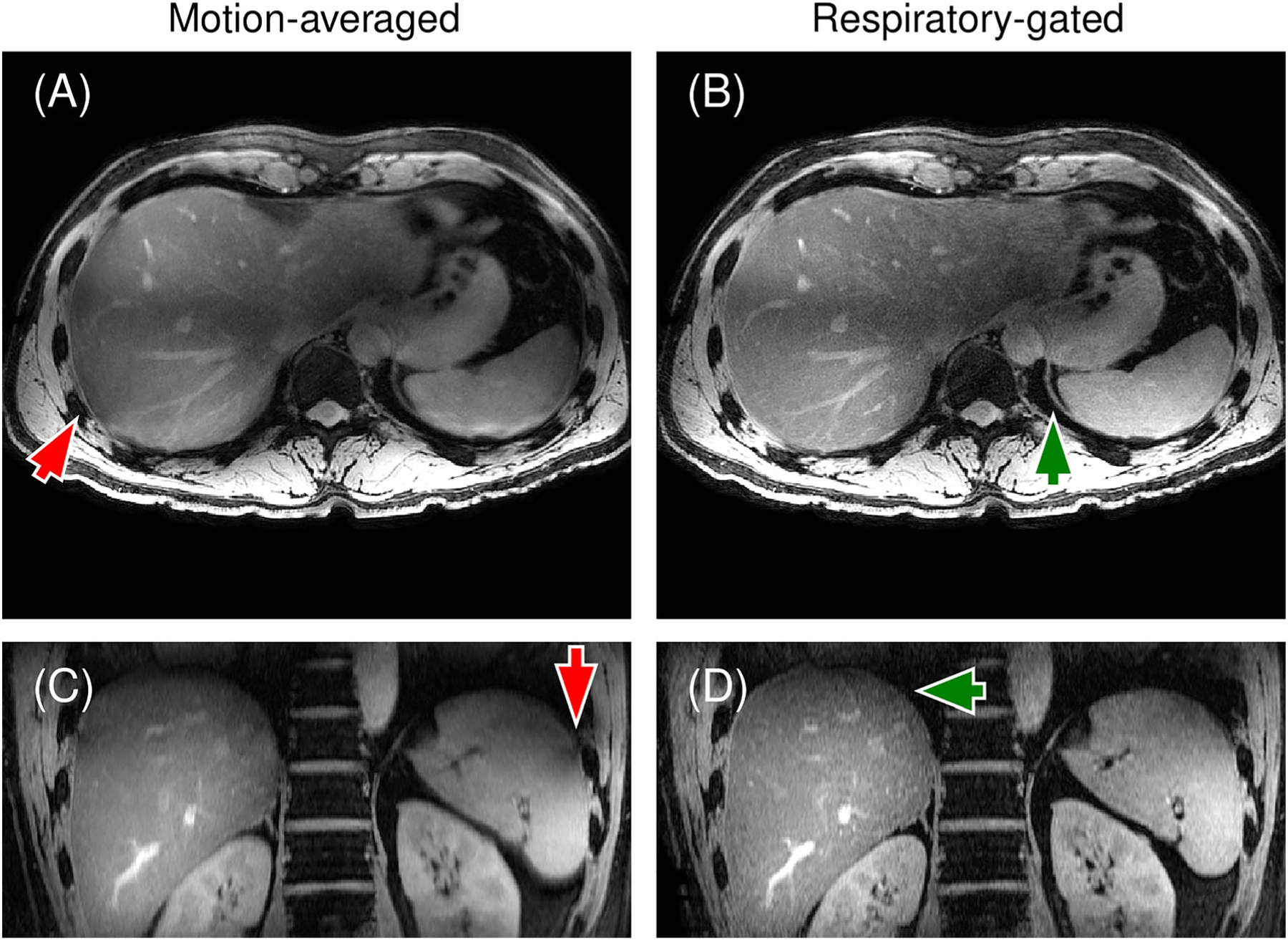
Comparison of (A and C) Motion-averaged, and (B and D) Respiratory-gated reconstructions of liver scan data. Respiratory gating resulted in sharp depictions of the diaphragm (arrow in (B)) and the liver dome (arrow in (D)). The uncorrected images (A) and (C) show large losses of signal near the diaphragm (arrows in (A) and (C)), which was attributed to phase errors due to translational motion.

**FIGURE 7:**
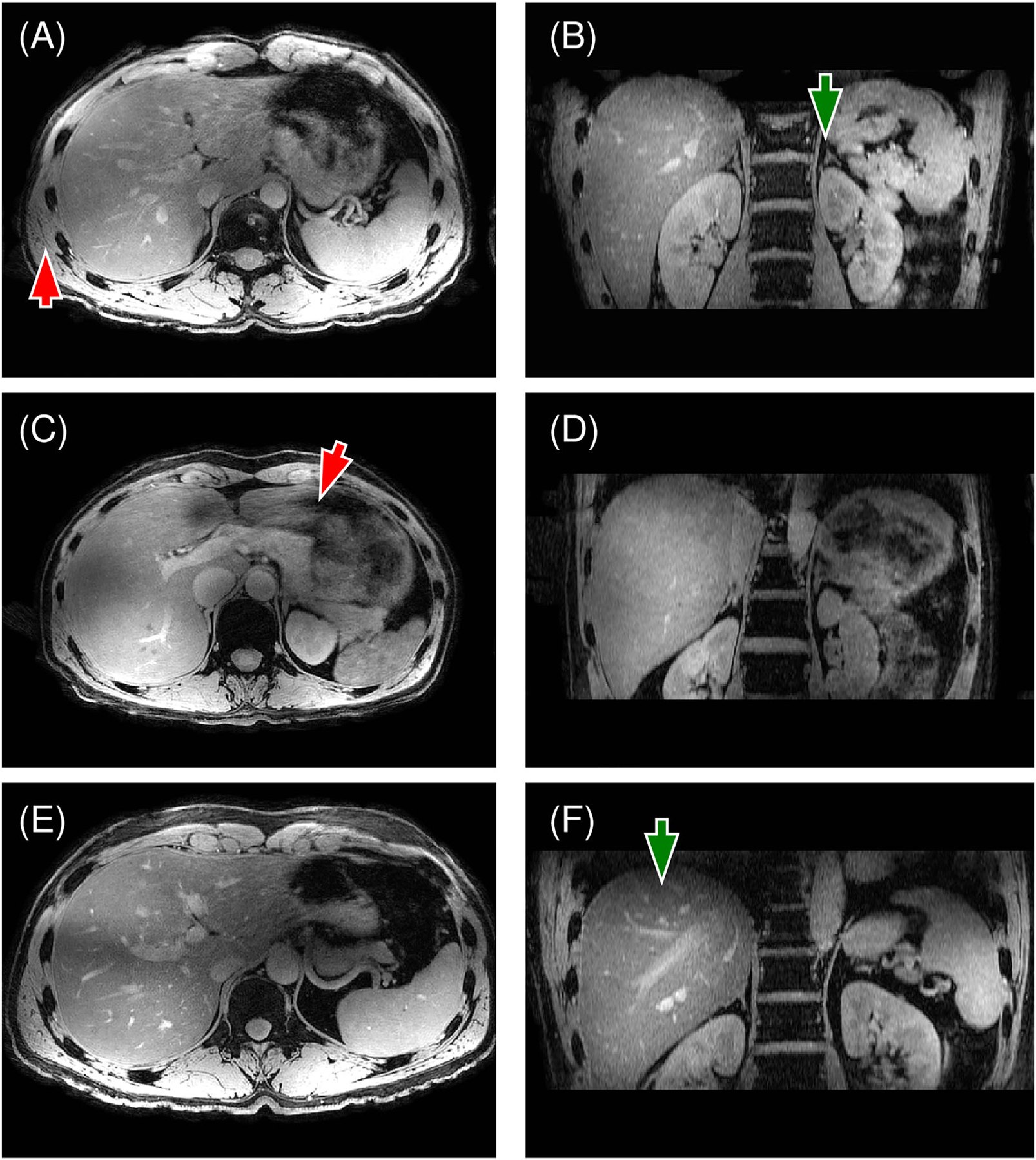
(A–F) Axial and coronal views of the liver images of three volunteers. The images show enhancement of the vessels without the use of an external contrast agent. The reconstructions demonstrated an effective ${\text{B}}^{+}$ shim with only minor fluctuations in signal strength at the liver in axial views (arrow in (A)). The coronal views display sharp depictions of the adrenal glands (arrow in (B)) and the vessels (arrow in (F)). Some streaking artifacts were visible and originated from the abdominal cavity (arrow in (C)).

Respiratory gating with a 40% data acceptance window increased the sharpness of the image reconstructions at the cost of reduced SNR (Figure 6). Respiratory-gated image reconstructions increased vessel sharpness and visibility of the diaphragm in the final exhale respiratory phase images. Furthermore, these images had reduced signal loss at the liver dome resulting from phase errors and from imaging slices moving outside of the original image plane.^53^ Some lower signal levels were seen at the liver dome caused by residual motion and the gradual slopes of the slab edges (Figure 7). The sharpness of the slab edges was restricted due to the low number of pulse lobes of the excitation pulses, which resulted from the short time windows between individual RF pulses.

**FIGURE 8:**
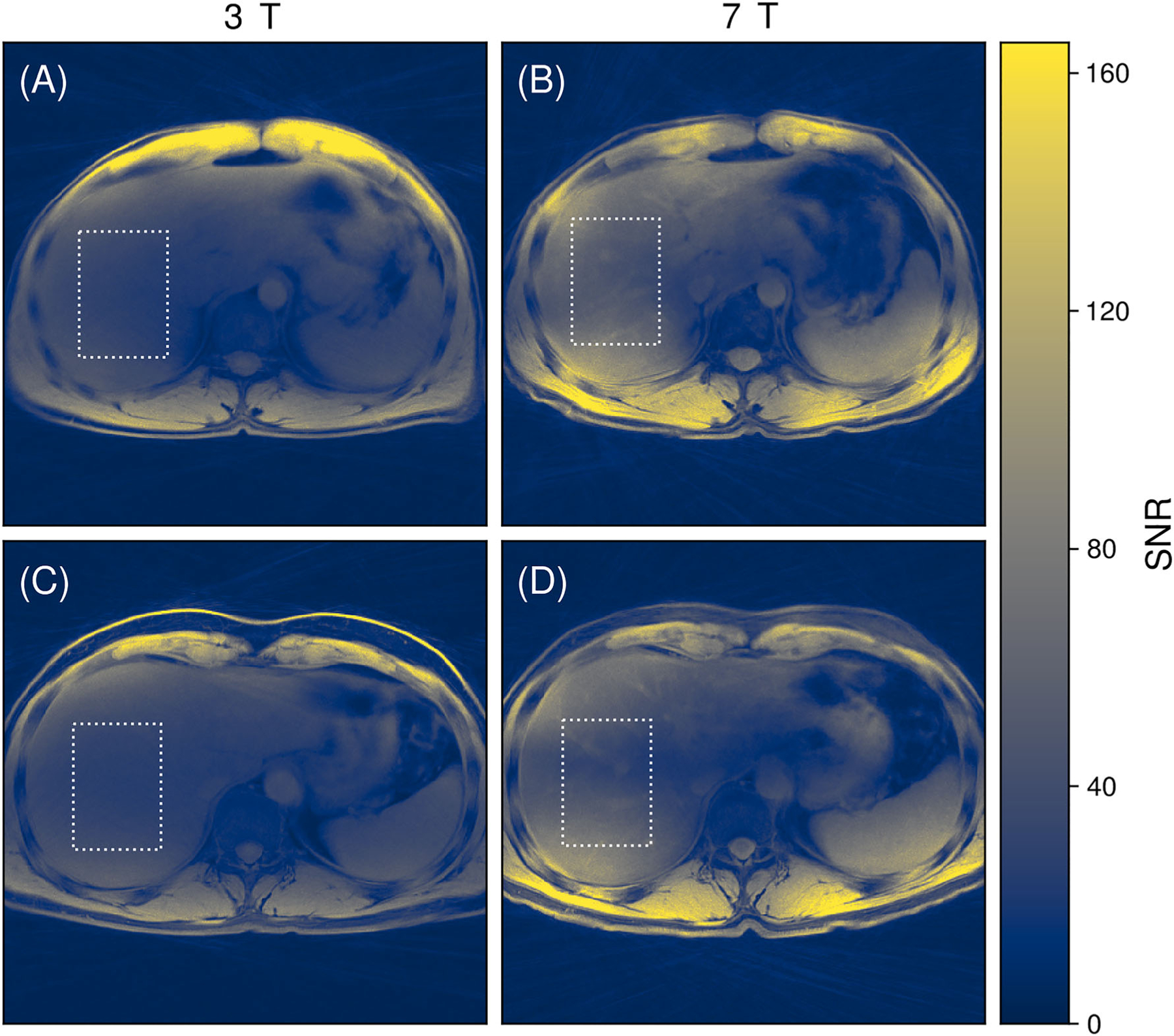
(A–D) Axial views of SNR maps averaged across 20 liver slices (or 2-cm slice thickness), generated through the “pseudo multiple replica” method. Each row corresponds to data from a different volunteer, with dotted outlines delineating regions of interest for indicative mean SNR calculations. In general, SNRs were superior in 7-T acquisitions, except in areas near the anterior side, where the dense body coil receiver arrays of the 3-T system exhibited better performance compared with the fractionated dipole antennas of the 7-T system. SNR, signal-to-noise ratio.

In the paired comparison with 3 T in two volunteers, 7-T acquisitions showcased SNR increases of 77% (31 ± 7 vs. 54 ± 13) and 58% (30 ± 5 vs. 48 ± 16) within the selected ROIs near the FOV center (Figure 8). The 3-T acquisitions displayed higher SNRs anteriorly, close to the high-density body coil arrays. The images used for qualitative comparison between 3 and 7 T are displayed side-by-side for all axial plane slices for both volunteers in the supporting information (Data S1 and Video S1). Compared with 7-T, the 3-T data showed an improved performance of subcutaneous fat suppression, increased image homogeneity, and reduced artifacts at the liver dome. On the other hand, similar streak artifacts were observed at 3 T near the abdominal cavity, despite an increase in the number of projection angles, as well as a greatly reduced CNR of the liver vessels.

### 3.2 │ Radiological evaluation

Image quality of the kidney MR scans was rated as good to excellent in the radiological image quality evaluation, along with nonexistent or insignificant impairment due to ${\text{B}}_{1}$ and motion artifacts. Delineation of the kidney vessels was rated as good to excellent with axial, coronal, and sagittal median scores and IQRs of 5.0 (4.3–5.0), 4.0 (4.0–5.0), and 4.0 (4.0–4.8), respectively (Table 2). Portrayal of the adrenal glands exhibited good to excellent image quality with medians and IQRs of 5.0 (4.3–5.0) in axial, 4.5 (4.0–5.0) in coronal, and 4.0 (4.0–4.0) in sagittal planes. Depiction of the ureter received similarly high ratings (axial, coronal, and sagittal scores of 5.0 [4.3–5.0], 4.5 [4.0–5.0], and 4.0 [4.0–5.0], respectively). The higher scores given to the axial views compared with the coronal and sagittal views reflected the higher in-plane spatial resolution in this orientation. Image impairments due to motion artifacts and ${\text{B}}_{1}$ were rated overall as nonexistent or insignificant for the kidney MR images, with values of 3.0 (3.0–3.0) and 3.0 (3.0–3.0), respectively. However, considering the healthy BMI range of 19.6–24.1 kg/m^2^ of the included subjects, limited signal strength in the FOV center may become more prominent at larger BMI values.

Overall, the quality of the liver image data was rated as moderate to excellent, with a moderate level of impairment resulting from motion artifacts. Regarding the liver vessels, median scores and IQRs of 4.0 (4.0–5.0), 3.0 (3.0–4.0), and 3.0 (3.0–4.0) were observed in the axial, coronal, and sagittal planes, respectively. Liver vessels and arteries showed enhancement without the use of a contrast agent (Figure 7). Meanwhile, on average, the diaphragm exhibited good to excellent portrayal, with scores of 5.0 (4.3–5.0) in axial, 4.0 (4.0–4.0) in coronal, and 4.0 (4.0–4.8) in sagittal orientations. Higher scores for the axial plane corresponded to the increased spatial resolution in the axial orientation compared with the coronal and sagittal views. Image impairments were deemed insignificant for ${\text{B}}_{1}$ inhomogeneity-related impairment (3.0 [3.0–3.0]) and moderate for impairment stemming from motion (2.0 [1.3–2.0]). These assessments showcased the general effectiveness of the TIAMO $B_{1}^{+}$ shim, along with some persisting streak artifacts due to residual motion and susceptibility artifacts at air–tissue interfaces. These streaking artifacts originated near the abdominal wall but were visible throughout the image, depending on the amount of motility of the intestinal system and air in the bowel lumen and stomach. An animation of all the axial images of the liver data for three volunteers is available for viewing in the supporting information (Data S1 and Video S2). The animation displays a homogeneous signal distribution, with streaking artifacts persisting primarily near the liver dome, with signal hyperintensities remaining on the posterior side. The latter is attributable to the lack of an integrated body RF coil on the 7-T system, so that a reference image for final image normalization is not available.

## 4 │ DISCUSSION

This work demonstrates an acquisition method for UHF free-breathing MRI of the upper abdomen at a high spatial resolution of 0.8 × 0.8 × 2.0 mm^3^ using a respiratory-gated radial stack-of-stars acquisition and TIAMO $B_{1}^{+}$ shimming. The TIAMO technique resulted in a nearly homogeneous signal distribution, lipid signal contamination was effectively avoided using water excitation with a binomial pulse, and motion artifacts were reduced through respiratory gating using FID navigators. In a direct comparison with 3-T MRI, the proposed method showcased increased SNRs, despite the overall development of body coil arrays at 7 T being at a relatively early stage.

The image quality of both the kidney and liver MRI data was rated highly by two radiologists in a qualitative evaluation. Image degradation due to ${\text{B}}_{1}$ inhomogeneity was generally viewed as insignificant, underscoring the effectiveness of the TIAMO $B_{1}^{+}$ shim across large FOVs. In earlier work, results of noncontrast-enhanced renal angiography without the use of the TIAMO technique were limited by residual B_1_ transmit inhomogeneity of RF shimming over a large FOV covering two kidneys.^15^ According to the radiological evaluation, these problems were deemed minimal in our volunteer cohort, with only minor residual ${\text{B}}_{1}$ shading in the MR images. Conversely, reconstructions of the radially sampled k-space data showed streaking artifacts on the anterior side of some volunteers. These streaking artifacts were also observed at 3 T and probably resulted from residual respiratory, cardiac, or intestinal motion, a slight undersampling factor, or from susceptibility differences, and were considered more problematic for the liver than for the kidney MR images. This difference may be related to the kidneys’ retroperitoneal location somewhat limiting possible motion, compared with the liver, which is located in the abdominal cavity and near the diaphragm. However, streaking artifacts resultingfrom intestinal motion can be greatly reduced in a clinical MRI setting when using antiperistaltic drugs to inhibit intestinal motion.^65^ Small angular increments between the readout lines of the two complementary TIAMO modes, similar to the use of small blip gradients in radial multiecho MRI,^66^ could similarly result in reduced streaking with minimal increase in sensitivity to eddy current artifacts.^42^

The high ratings for image quality highlighted the potential of 7-T abdominal radial stack-of-stars MRI for clinical application. For example, the reconstructed images showed excellent visibility of blood-supplying vessels in the liver due to blood hyperintensity without the use of a contrast agent. Similar enhancements of the arterial vasculature have been observed before in several studies including 7-T kidney,^67^ liver,^68^ and intracranial^69–71^ MRI. It has been suggested that this effect is linked to in-flow effects or differences in T_1_ relaxation times, which have been reported to increase substantially at 7 T for both blood and hepatic tissue. Because in our case vessel enhancements were observed throughout the slab and not only at its edges, we deem it unlikely that vessel enhancement resulted from in-flow effects. Instead, the unique contrast is more likely to be related to the relatively low flip angle applied (≈ 2.5^°^) and the differences in T_1_ and T_2_^*^ relaxation times. The resulting enhancements seen within the current study are in line with previous findings, suggesting that 7-T MRI may have unique MR angiography applications for patients who have contraindications to gadolinium contrast agents.^14^

Our method holds promise for various other clinical applications. An improved spatial resolution can significantly aid in identifying pancreatic ductal adenocarcinoma at earlier stages, a disease whose detection sensitivity drops significantly for tumors smaller than 2 cm.^72,73^ Measurements of decreased pancreatic volumes utilizing T_1_-weighted GRE sequences have been shown to be predictors of endocrine dysfunction.^74^ Precision in measuring pancreatic volume changes has potential significance in guiding clinical decisions for children affected by early-onset type 1 diabetes, which is one of the most prevalent chronic autoimmune diseases in childhood.^75^ Furthermore, an accurate depiction of the (change in) morphology of upper abdominal lymph nodes, which may have short axis diameters as low as 1.5 mm, is traditionally used as an indicator of metastatic spread in oncology, directly impacting patient prognosis and treatment planning.^17,76,77^ Currently, distinguishing between benign and malignant nodes relies heavily on their morphology, demanding a high spatial resolution, preferably in all three spatial dimensions.

A straightforward extension of the proposed sequence would be to acquire multiple gradient echoes, which would allow for the generation of high-resolution T_2_^*^-weighted images. Prior research demonstrated that signal decay levels in abdominal lymph nodes postadministration of an ultrasmall superparamagnetic iron oxide (USPIO) contrast agent can improve the detection of prostate cancer metastases in these nodes.^78,79^ Extending this generic mechanism of action to cancer of the esophagus, pancreas, stomach, or kidneys could aid in detecting early metastatic disease.^19,76^ Next to this, in kidney transplant patients, a diminished function of allografts is expected to correlate with reduced medullary R_2_^*^ values due to sodium accumulation.^13^ Challenges were encountered in measuring differences in cortical R_2_^*^ values because of relatively low SNRs, a limitation that could potentially be mitigated by employing an UHF strength.

In the current work, we used a radial stack-of-stars sequence because of its inherent resilience to motion artifacts, in particular when compared with a Cartesian encoding scheme. To the best of our knowledge, this is the first illustration of a free-breathing radial acquisition for upper abdominal MRI at UHF, as well as its first evaluation by expert radiologists. However, 2D radial cross-sectional imaging during breath-holds has been showcased before using an MR fingerprinting sequence.^80,81^ Also, in a recent study on cardiac imaging at 7 T, a successful demonstration of 3D motion-compensated MRI was achieved using a radial phase-encoding (RPE) sequence.^82^ Compared with the radial acquisition described in this study, the RPE sequence exhibits a reduced sensitivity to eddy currents and gradient delays. Because the k-space center is not sampled at every pulse repetition time, the RPE acquisition lacks the intrinsic robustness to motion artifacts characteristic of the radial stack-of-stars sequence.^83^ Additionally, the smaller ROI in cardiac imaging enabled the efficient use of a single static $B_{1}^{+}$ shim, removing the necessity to incorporate multiple TIAMO modes. Additionally, the authors utilized a Dixon approach for cardiac fat-water imaging, enabled by a triple-echo acquisition to correct for main field inhomogeneity. A similar fat-water separation in postprocessing could present a feasible alternative to the water excitation applied in this work, considering that the latter was shown to be limited by SAR constraints, as indicated by the flip angle maps obtained through the 3DREAM sequence.

Our current implementation of the radial stack-of-stars sequence employed a binomial pulse for water excitation as a measure to mitigate off-resonance blurring of lipid signals. Benefiting from the large lipid-water chemical shift of approximately 1 kHz at 7 T, the binomial pulse was played out significantly faster than at 3 T, improving the scan efficiency. The fixed interpulse durations, determined by the chemical shift, prevent prolonging the subpulses to reduce the peak $B_{1}^{+}$ . Consequently, the subpulses were relatively short, resulting in elevated peak RF power for a given flip angle. To mitigate this RF energy constraint, a small bandwidth-time product of 7 was utilized, and bipolar instead of monopolar gradients were used for slab selection. The bipolar gradients avoided having to add rephasing gradients between the RF subpulses, thereby allowing longer periods of RF transmission during the composite pulse. This reduced the SAR constraint by lowering the peak $B_{1}^{+}$ amplitude during transmission by 59%. Theoretically, the bipolar variant could lead to less efficient lipid suppression along the slab axis than its monopolar counterpart, because of the alternating direction of the chemical shift displacement of lipids between subpulses.^49^ In practice with the used pulse bandwidths, this effect appeared insignificant in our results.

The described acquisition strategy led to long scan times of almost 9 and 14 min for the kidney and liver scans, respectively. One reason for these long acquisition times was to investigate the achievable image quality without the added complexity of accelerated reconstruction techniques such as parallel imaging or compressed sensing. To decrease the acquisition time, the reconstruction strategy could be adapted to exploit the favorable acceleration properties of the TIAMO method. The original description of TIAMO showed that two complementary ${\text{B}}^{+}$ modes could be viewed as forming virtual receive elements.^34^ The virtual receive elements allowed superior performance of parallel imaging algorithms, thereby allowing TIAMO with a minimal cost in either acquisition time or SNR. Parallel imaging could be incorporated directly, such as in generalized autocalibrating partially parallel acquisitions (GRAPPA),^84^ or as an element of a compressed sensing algorithm.^85^ Compared with an acquisition method utilizing a single excitation mode, the incorporation of virtual receivers is likely to enable faster imaging through the utilization of larger undersampling factors.^86^

A limitation of the present study is the absence of calculated actual flip angles per volunteer. Given that relative $B_{1}^{+}$ maps suffice for the TIAMO shim optimization, the acquisition of absolute $B_{1}^{+}$ maps was not pursued. Additionally, recent work has suggested that the $B_{1}^{+}$ maps could show slight variation throughout the respiratory cycle.^47^ Therefore, the accuracy of the $B_{1}^{+}$ mapping is expected to improve by acquiring the calibration data through the use of a respiratory-corrected sequence as well, or during breath-holds. Finally, the $B_{1}^{+}$ calibration currently requires the manual selection of an ROI within a single offline reconstructed image. However, in our experience, this calibration is robust to minor changes within ROI selection and, therefore, will probably be replaced by an automatic segmentation or body mask in future applications.

## 5 │ CONCLUSION

This work has demonstrated that free-breathing abdominal MRI at 7 T is feasible, robustly providing high image quality at high spatial resolutions through the use of a radial stack-of-stars sequence, with an increased SNR in comparison with 3-T measurements. The radial stack-of-stars sequence was combined with dual-mode phase-only TIAMO $B_{1}^{+}$ shims to reconstruct respiratory-gated images with nearly homogeneous signal distributions. The qualitative evaluation of experimental data acquired in seven volunteers by two experienced radiologists demonstrated high image quality, with only minor impairments by ${\text{B}}_{1}$ inhomogeneity and motion artifacts. Overall, this research establishes a foundation for diverse radial stack-of-stars imaging methods in the upper abdomen at 7 T, expanding its use in this body region to previously unexplored clinical and research applications.

## Supplementary Material

Video 1

Video 2

Data S1

Fig 1

## Figures and Tables

**TABLE 1:** Sequence parameters for the 2D Cartesian FLASH $B_{1}^{+}$ calibration scan, as well as the radial stack-of-stars kidney and liver acquisitions. The 7-T kidney and liver MR acquisitions differed only in the number of acquired partitions and the positions of the slabs.

Parameter	7-T $B_{1}^{+}$ calibration	7-T kidneys	7-T liver	3-T liver
TE (ms)	2.60	3.00	3.00	4.0
TR (ms)	20.0	6.25	6.25	8.31
Base resolution	128	384	384	384
FOV (mm^2^)	384 × 384	320 × 320	320 × 320	320 × 320
Readout bandwidth (Hz/px)	590	390	390	380
Pixel size (mm^2^)	3.0 × 3.0	0.8 × 0.8	0.8 × 0.8	0.8 × 0.8
Slice thickness (mm)	5.0	1.0	1.0	1.0
Slice resolution (%)	100	50	50	50
Acquired partitions	1	42	66	66
Slice oversampling (%)	N/A	7.7	10.0	10.0
Slice partial Fourier	N/A	3/4	3/4	3/4
Radial views	N/A	1000	1000	1500
Orientation	Axial	Axial	Axial	Axial
Acquisition time (min:s)	0:20	8:45	13:45	13:44
Actual flip angle (^°^)	N/A	≈2.5	≈2.5	5

Abbreviations: FLASH, fast low angle shot; FOV, field of view; MR, magnetic resonance; TE, echo time; TR, repetition time.

**TABLE 2:** Median scores and interquartile ranges for each of the evaluated Likert-scale factors in the radiological image quality evaluation.

	Ratings MRI kidneys			Ratings MRI liver	
	Vessels	Proximal ureter	Adrenal glands	Vessels	Diaphragm
Transversal	5.0 (4.3–5.0)	5.0 (4.3–5.0)	5.0 (4.3–5.0)	4.0 (4.0–5.0)	5.0 (4.3–5.0)
Coronal	4.0 (4.0–5.0)	4.5 (4.0–5.0)	4.5 (4.0–5.0)	3.0 (3.0–4.0)	4.0 (4.0–4.0)
Sagittal	4.0 (4.0–4.8)	4.0 (4.0–5.0)	4.0 (4.0–4.0)	3.0 (3.0–4.0)	4.0 (4.0–4.8)
${\text{B}}_{1}$ inhomogeneity	3.0 (3.0–3.0)			3.0 (3.0–3.0)	
Motion artifacts	3.0 (3.0–3.0)			2.0 (1.3–2.0)	

Abbreviation: MRI, magnetic resonance imaging.

## Data Availability

The data that support the findings of this study are available from the corresponding author upon reasonable request.

## Abbreviations:

3DREAM: 3D dual refocusing echo acquisition mode
FID: free induction decay
FLASH: fast low angle shot
GRAPPA: generalized autocalibrating partially parallel acquisitions
GRE: gradient-recalled echo
iNUFFT: inverse nonuniform fast Fourier transform
IQR: interquartile range
pTx: parallel transmit
ROI: region of interest
RPE: radial phase encoding
TIAMO: time-interleaved acquisition of modes
UHF: ultrahigh field
USPIO: ultrasmall superparamagnetic iron oxide
