# Supplementary material for: Free-breathing high-resolution respiratory-gated radial stack-of-stars magnetic resonance imaging of the upper abdomen at 7 T
Source: NMR Biomed. Author manuscript; Available in PMC 2025 Apr 15. (PMC11998609; doi:10.1002/nbm.5180)
Supplement: Data S1 [file NIHMS2064098-supplement-Data_S1.docx]

**Captions for Online Videos**

**Supplementary Video 1.** Animations of all axial images of the 3T and 7T comparison scans of two volunteers. Compared to 7T, the 3T data showed an improved performance of subcutaneous fat suppression, increased image homogeneity, and reduced artifacts at the liver dome. On the other hand, similar streak artifacts were observed at 3T near the abdominal cavity despite an increase in the number of projection angles, as well as a greatly reduced contrast-to-noise ratio of the liver vessels.

**Supplementary Video 2.** Animations of all axial images of the liver data of three volunteers. The images display the effectiveness of TIAMO pTx throughout the FOV with only minor variations in signal intensity. The application of the binomial pulse excited the water signal efficiently, with only minor residual subcutaneaous lipid signal. However, some streaking artifacts can be observed on the anterior side, as well as signal loss due to spatial variation in magnetic susceptibility.
