# Supplementary material for: Free-breathing high-resolution respiratory-gated radial stack-of-stars magnetic resonance imaging of the upper abdomen at 7 T
Source: NMR Biomed. Author manuscript; Available in PMC 2025 Apr 15. (PMC11998609; doi:10.1002/nbm.5180)
Supplement: Fig 1 [file NIHMS2064098-supplement-Fig_1.docx]

**Supporting Figures**


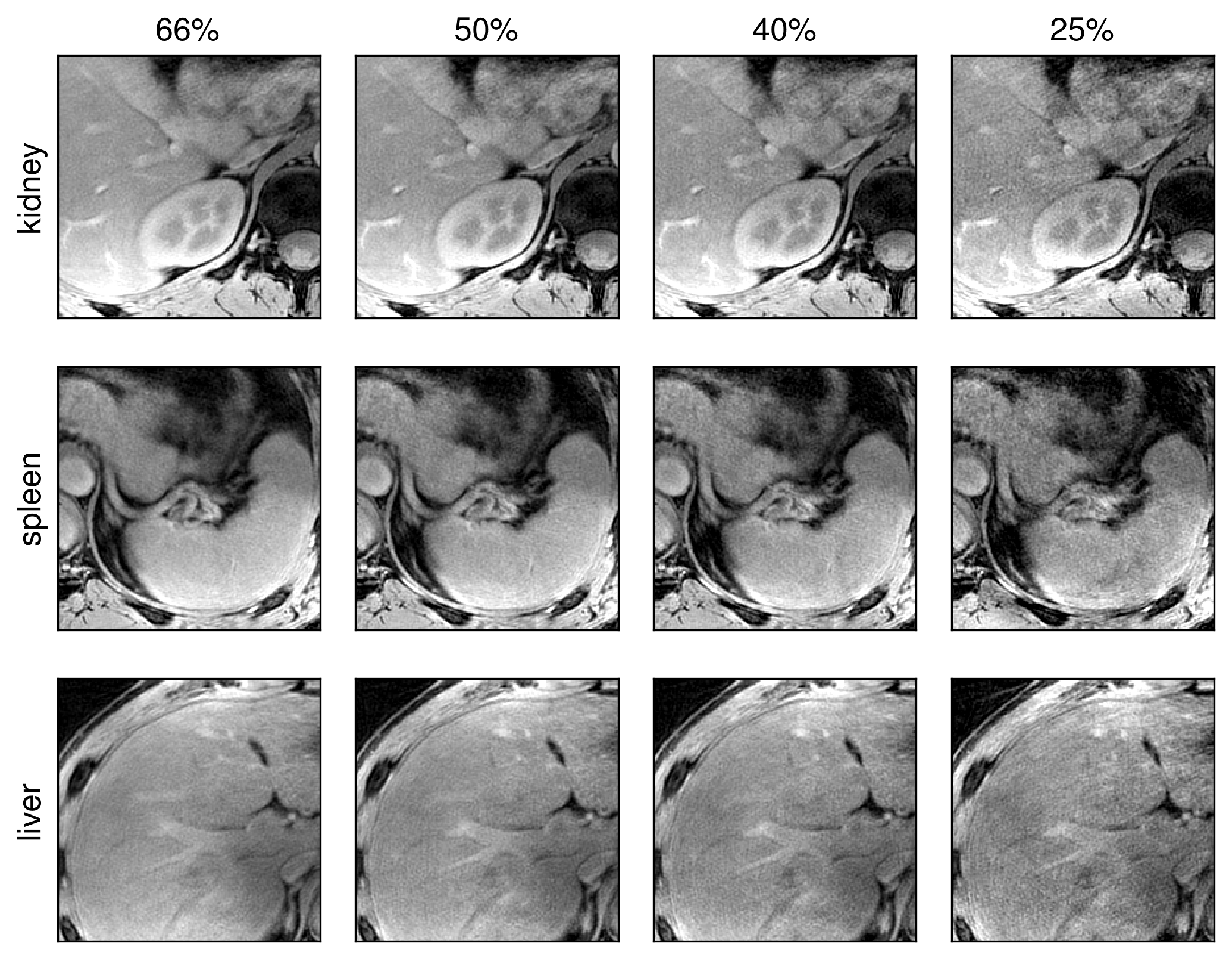


**Figure 1.** Four separate respiratory-gated reconstructions of the 7T liver data of a single volunteer using 66%, 50%, 40%, and 25% of the total k-space data. Images are displayed for three different regions-of-interest: kidney, spleen, and liver. A value of 40% was determined to be a favorable trade-off between SNR and residual motion blur.
